# Role of PFKFB3-driven glycolysis in sepsis

**DOI:** 10.1080/07853890.2023.2191217

**Published:** 2023-05-18

**Authors:** Min Xiao, Dadong Liu, Yao Xu, Wenjian Mao, Weiqin Li

**Affiliations:** a Department of Critical Care Medicine, Affiliated Jinling Hospital of Nanjing Medical University, Nanjing, Jiangsu, China; b Department of Critical Care Medicine, Affiliated Jinling Hospital of Nanjing University, Nanjing, China; c Center of Severe Acute Pancreatitis (CSAP), Jinling Hospital, Nanjing, Jiangsu, China

**Keywords:** sepsis, glycolysis, PFKFB3

## Abstract

Sepsis is still the leading cause of death as a result of infection. Metabolic disorder plays a vital role in sepsis progression. Glycolysis intensification is the most characteristic feature of sepsis-related metabolic disorders. The enzyme 6-phosphofructo-2-kinase/fructose-2,6-bisphosphatase 3 (PFKFB3) is a critical engine that controls the rate of glycolysis. Recent studies have revealed that sepsis accelerates the rate of PFKFB3-driven glycolysis in different cells, including macrophages, neutrophils, endothelial cells and lung fibroblasts. Furthermore, increased PFKFB3 is closely related to the excessive inflammatory response and high mortality in sepsis. Interestingly, inhibition of PFKFB3 alone or in combination has also shown great potential in the treatment of sepsis. Therefore, an improved understanding of the canonical and noncanonical functions of PFKFB3 may provide a novel combinatorial therapeutic target for sepsis. This review summarizes the role of PFKFB3-driven glycolysis in the regulation of immunocyte activation and nonimmune cell damage in sepsis. In addition, we present recent achievements in the development of PFKFB3 drugs and discuss their potential therapeutic roles in sepsis.

**KEY MESSAGE**
1. Sepsis induces high expression of PFKFB3 in immunocytes and nonimmune cells, thereby enhancing cellular glycolytic flux.
2. PFKFB3-driven glycolysis reprogramming is closely related to an excessive inflammatory response and high mortality in sepsis.
3. Inhibition of PFKFB3 alone or in combination provides a novel combinatorial therapeutic target for sepsis.

## Introduction

Sepsis is the leading cause of death and is defined as life-threatening organ dysfunction caused by the dysregulated host response to infection [1–3]. Inflammatory damage of tissues and organs caused by immune system overreaction is an important mechanism of high mortality of sepsis [4]. Immunocyte activation is the central engine of the immune system to fight insults caused by invading pathogens [5]. Nonimmune cell damage is the fundamental mechanism of sepsis-induced organ inflammatory damage [6]. Investigating the mechanisms of immunocyte activation and nonimmune cell damage is the focus and hotspot of sepsis research.

Current knowledge on sepsis indicates that glycolysis intensification is the most characteristic feature of sepsis-related metabolic disorders [7]. Glycolysis is a metabolic pathway that is catalyzed by multiple metabolic enzymes in the cytosol and is used by most human cells for energy generation [8]. In addition to maintaining cell growth and differentiation, glycolysis has been considered a key player in the inflammatory response [8–11]. Accumulating evidence has proven that increased glycolysis in immunocytes is closely related to the excessive inflammatory response in sepsis [7,12–14]. Interestingly, some well-executed experimental studies have revealed that pharmacological inhibition of glycolysis is associated with reduced mortality [15–17]. Therefore, efforts to understand how to regulate glycolysis reprogramming may provide clues for developing better therapeutics for sepsis.

**Figure 1.**
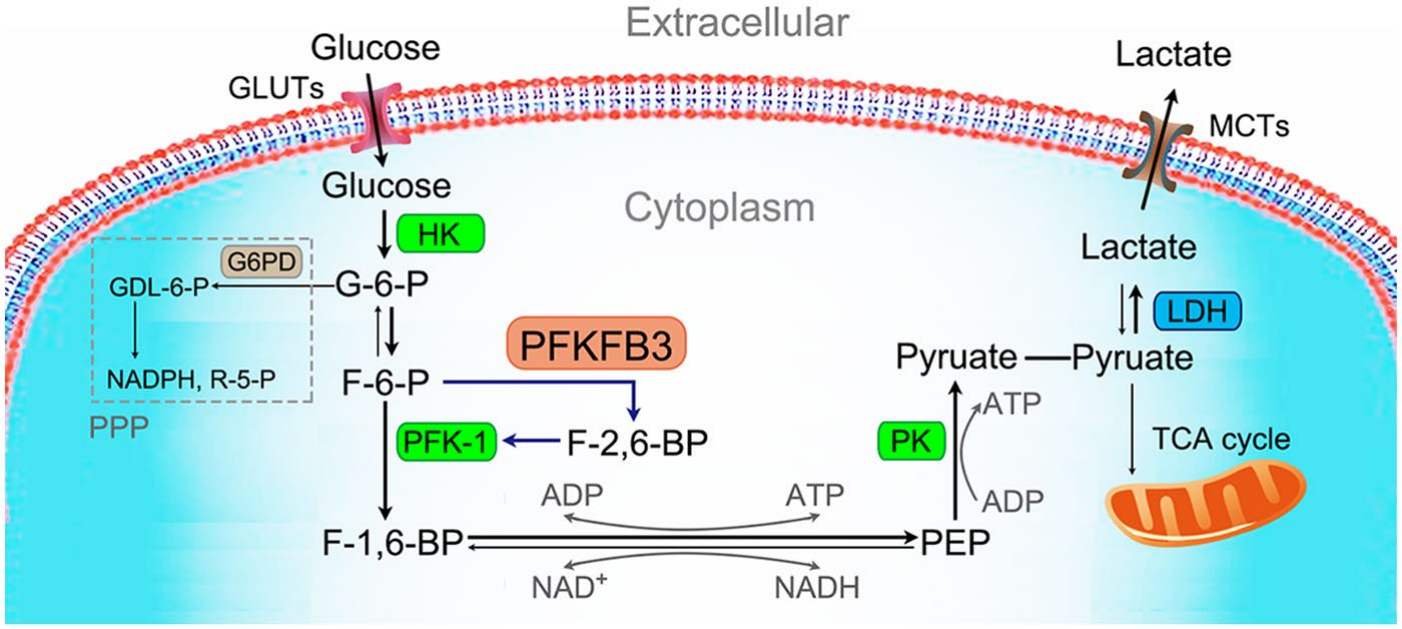
Glycolytic cycle. Glycolysis is the ancient metabolic pathway that converts glucose into pyruvate. Glucose transporters (GLUTs), the transfer proteins located on the cell membrane, uptake extracellular glucose. Cytoplasmic glucose is phosphorylated by hexokinase (HK), the first rate-limiting enzyme of glycolysis, to form glucose 6-phosphate (G-6-P). G-6-P is then rearranged into fructose 6-phosphate (F-6-P) by glucose phosphate isomerase. F-6-P is then irreversibly converted to fructose-1,6-bisphosphate (F-1,6-BP) under the catalysis of phosphofructokinase-1 (PFK-1), the main rate-limiting enzyme of glycolysis. Fructose-2,6-bisphosphate (F-2,6-BP), a product of the reaction catalyzed by 6-phosphofructo-2-kinase/fructose-2,6-bisphosphatase 3 (PFKFB3), is the most potent positive allosteric effector of PFK-1. Then, following a series of reversible enzymatic reactions, F-1,6-BP is converted to phosphoenolpyruvate (PEP). In addition, nicotinamide adenine dinucleotide phosphate (NADH) and triphosadenine (ATP) are generated in these reactions. Finally, PEP is phosphorylated by pyruvate kinase (PK), the third rate-limiting enzyme of glycolysis, to form pyruvate and a molecule of ATP. In the absence of oxygen, pyruvate is converted to lactate under the catalysis of lactate dehydrogenase (LDH). Lactate is then transported extracellularly through monocarboxylate transporters (MCTs). In the case of oxygen, pyruvate enters the mitochondria for the tricarboxylic acid (TCA) cycle.

Recently, 6-phosphofructo-2-kinase/fructose-2,6-biphosphatase 3 (PFKFB3), a bifunctional enzyme regulating glycolysis, has been brought to the forefront of immune metabolism research. It can accelerate glycolysis by modulating and maintaining the intracellular concentrations of fructose-2,6-bisphosphate (F-2,6-BP) to allosterically activate 6-phosphofructokinase-1 (PFK-1), the key rate-limiting enzyme of glycolysis (Figure 1) [18]. Recent studies have revealed that PFKFB3 is widely expressed in tissues and plays a vital role in the occurrence and metastasis of tumors, organ damage in diabetes mellitus, and angiogenesis [19–21]. Alterations in the levels of PFKFB3 have been reported in different sepsis-associated cells, such as macrophages [22], neutrophils [22], endothelial cells (ECs) [23] and lung fibroblasts [24]. Furthermore, increased PFKFB3 is associated with an excessive inflammatory response in sepsis. Thus, PFKFB3 has become a novel therapeutic target for inhibiting excessive inflammation in sepsis.

In the present review, we summarize the role of PFKFB3 in sepsis based on the latest studies in the field of PFKFB3 gene and protein. We also present recent achievements in the development of PFKFB3 drugs and discuss their potential therapeutic role.

### General characteristics of PFKFB3

**Figure 2.**
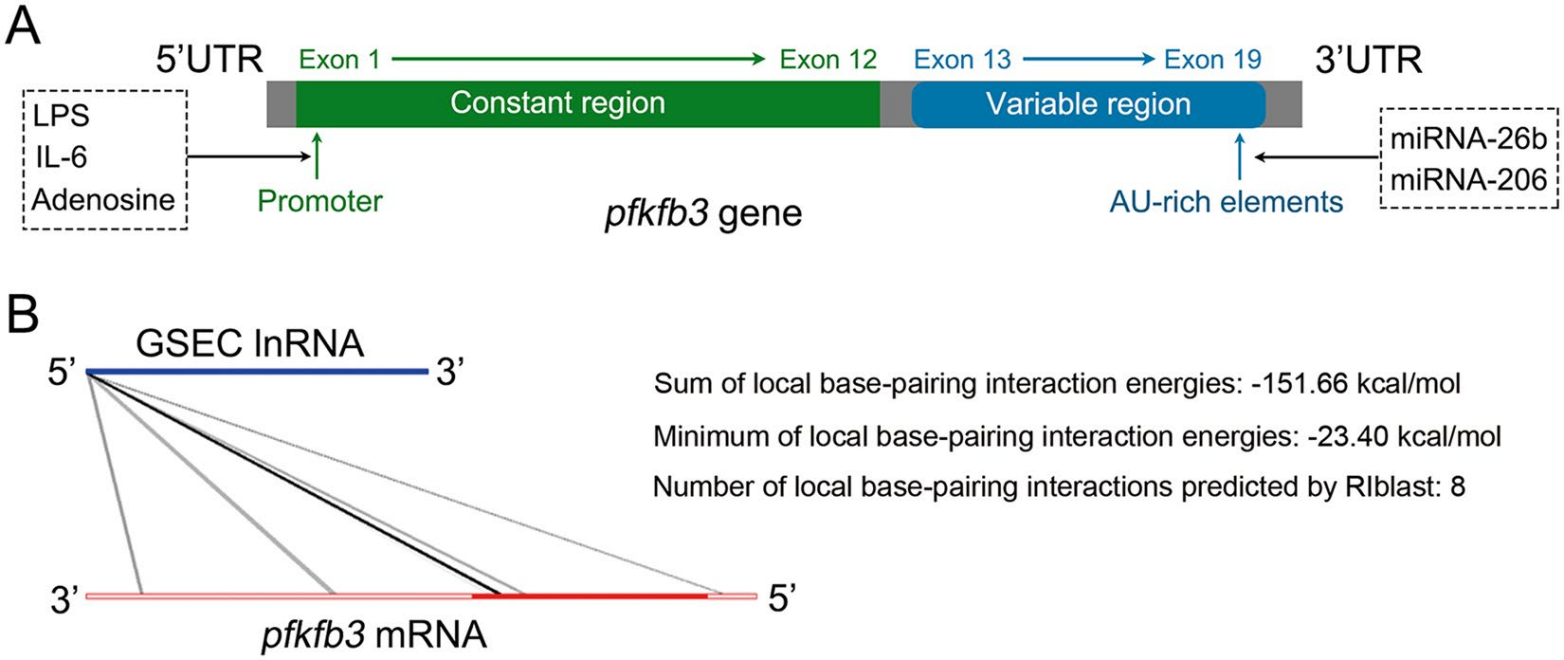
PFKFB3 gene. (A) Schematic of the coding sequence of the human *pfkfb3* gene that consists of 19 exons. (B) The LncRRIsearch website (http://rtools.cbrc.jp/LncRRIsearch/) predicted that GSEC might interact directly with PFKFB3. The potential interaction possibly involves 8 regions, including two 3′ untranslated regions (UTRs), five coding sequence (CDS) regions, and one 5’ UTR. The sum of the global base-pairing interaction is described as an image. The query RNA (GSEC) and the target RNA (PFKFB3) are represented as a blue line and a red line, respectively. Predicted interactions are displayed as gray or black lines between two RNAs. The color consistency indicates the strength of the interactions.

PFKFB3, a brain/placenta isoenzyme, is one of the four PFKFB isoforms and is encoded by the *pfkfb3* gene, which is located on the short arm of chromosome 10 at position p15.1 [25]. The gene is approximately 109.547 kb in length and contains at least 19 exons, which are subdivided into a constant region (containing 12 exons) and a variable region (containing 7 exons) (Figure 2(A)) [19,26–28]. In the 5′ untranslated region (5’UTR), the *pfkfb3* promoter contains multiple binding sites that can be stimulated by proinflammatory molecules, such as lipopolysaccharide (LPS), interleukin (IL)-6, and adenosine [28]. The 3’UTR of the *pfkfb3* gene contains multiple copies of the AU-rich sequence, which confers enhanced instability and translational activity [29]. Previous studies have reported that miRNA can reduce the rate of glycolysis by directly interacting with the 3’UTR of the *pfkfb3* gene [30,31]. In addition, lncRNA–RNA interaction analysis predicted that lncRNA GSEC might have a direct interaction with PFKFB3 (Figure 2(B)) [32]. This result was further proven by dual luciferase reporter assays in our previous study [22].

**Figure 3.**
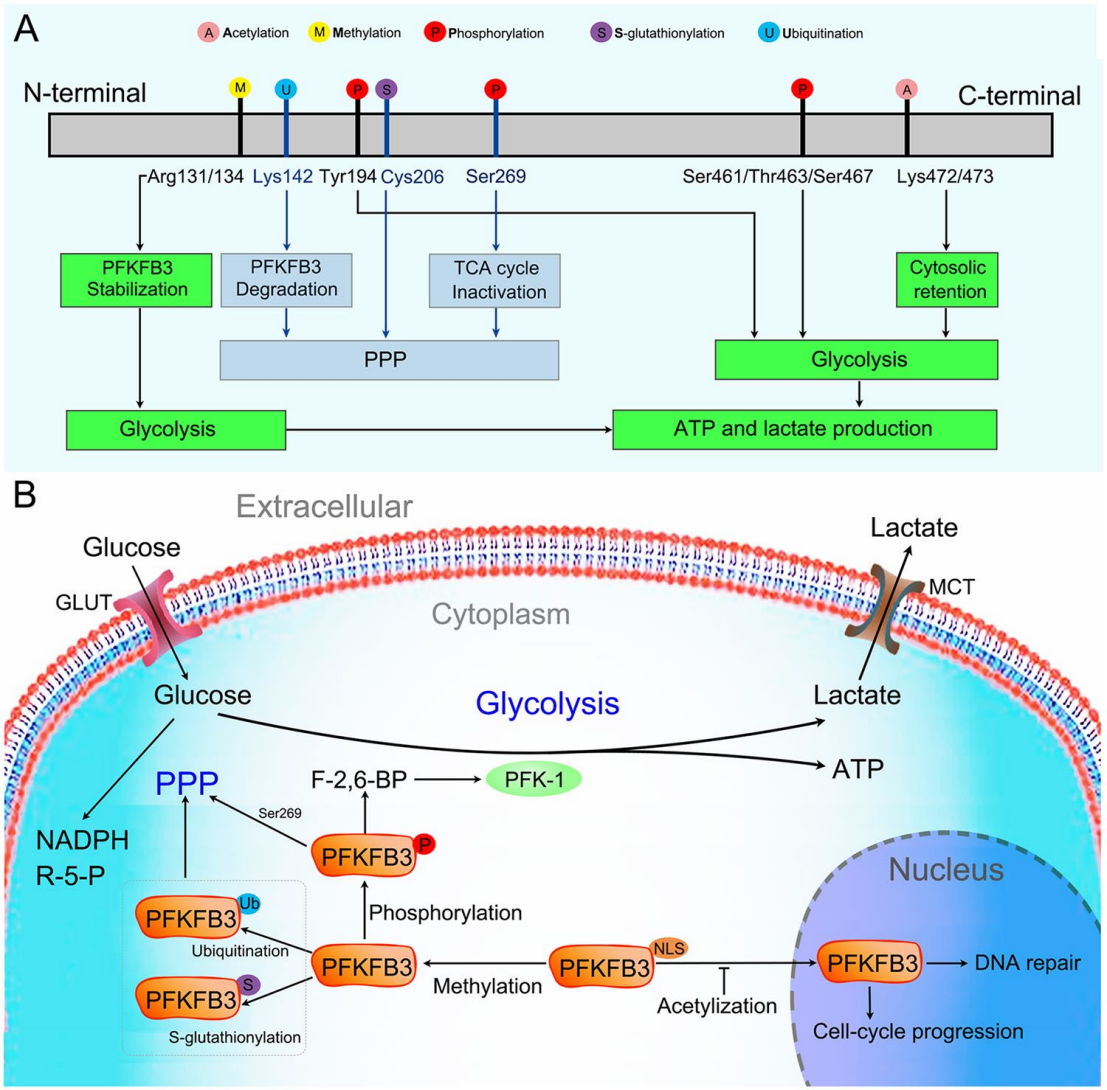
PFKFB3 protein. (A) Schematic of PFKFB3 protein sequences. PFKFB3 can be phosphorylated at Ser461 by adenosine monophosphate-activated protein kinase (AMPK), MAPK-activated protein kinase 2 (MK2), mammalian target of rapamycin 2 (mTOR2) and protein kinase C (PKC), at Tyr463/Ser467 by cyclin-dependent kinase 6 (CDK6), and at Tyr194 by c-Src. Phosphorylated PFKFB3 promotes glycolysis. Asymmetrical dimethylation of Arg131/134 stabilizes PFKFB3. Acetylation of Lys472/473 of PFKFB3 protein promotes cytosolic retention of PFKFB3. Both asymmetrical demethylation and acetylation promote glycolysis. Reduced PFKFB3 activity *via* ubiquitination of Lys142 by mitogen-activated protein kinase 14 (MAPK14), S-glutathionylation of Cys206 by reactive oxygen species (ROS) and phosphorylation of Ser269 by nuclear factor kappa-B kinase subunit beta (IKKβ) reduces flux into the oxidative arm of the pentose phosphate pathway (PPP). (B) PFKFB3 function in cells. The PFKFB3 protein is mainly located in the cytoplasm. In the cytoplasm, PFKFB3 is phosphorylated by a series of kinase proteins and promotes glycolysis by modulating and maintaining the production of fructose-2,6-bisphosphate (F-2,6-BP) to allosterically activate the glycolytic enzyme 6-phosphofructokinase-1 (PFK-1). Cytoplasmic PFKFB3 contains a nuclear localization signal (NLS), allowing it to traffic to the nucleus, where it participates in controlling cell cycle progression and DNA repair in specific contexts. However, acetylation prevents NLS recognition and promotes PFKFB3 cytoplasmic retention. PFKFB3 S-glutathionylation and ubiquitination inactivate PFKFB3 and lead to a shift in glucose utilization from glycolysis to the PPP.

The PFKFB3 protein consists of 520 amino acids, has a predicted molecular weight of 60 kDa, and has a high kinase/phosphatase ratio (∼740:1), which favors metabolic flux in glycolysis [33–35]. Cell localization analysis showed that the PFKFB3 protein is mainly located in the cytoplasm, which is the main site of glycolysis [36–38]. Cytoplasmic PFKFB3 can be phosphorylated by different protein kinases (Figure 3(A)) [39–45]. Phosphorylated PFKFB3 allosterically activates PFK-1 through the production of F-2,6-BP and promotes increased cellular glycolytic flux (Figure 3(B)). In addition, cytoplasmic PFKFB3 contains a nuclear localization signal (NLS), allowing it to traffic to the nucleus where it participates in the control of cell cycle progression and DNA repair in specific contexts (Figure 3(B)) [36,46]. However, acetylation at Lys472/473 of the PFKFB3 protein prevents NLS recognition and promotes cytosolic retention of PFKFB3 (Figure 3). Similar to acetylation, methylation at Arg131/134 of PFKFB3 leads to stabilization of the PFKFB3 protein and promotes increased cellular glycolytic flux (Figure 3(A)).

In addition to glycolysis promotion, PFKFB3 has been reported to participate in the pentose phosphate pathway (PPP). In the presence of excessive reactive oxygen species (ROS), PFKFB3 is rapidly S-glutathionylated on Cys206, which inactivates PFKFB3 and leads to a shift in glucose utilization from glycolysis to the PPP (Figure 3(B)) [47]. This process allows cancer cells to use glucose to synthesize antioxidants, such as NAPDH and glutathione (GSH), and enables cells to escape the detrimental effects of excessive oxidative stress. Similar to S-glutathionylation, ubiquitin-mediated proteasomal degradation of PFKFB3 protein is an important factor in glycolysis inhibition and PPP activation (Figure 3(B)) [48–50].

Under physiological conditions, PFKFB3 is expressed at low levels in all tissues. A previous study indicated that the expression of PFKFB3 was significantly increased in macrophages after lipopolysaccharide (LPS) stimulation for 6 h [51]. In addition, the phosphorylation of PFKFB3 at serine 461 (Ser461) is significantly increased in long-term LPS-stimulated macrophages, which points to enhanced glycolytic flux and favors ATP generation [52]. In addition, PFKFB3 in the cytoplasm is rapidly increased and phosphorylated under hypoxic conditions, which contributes to increased glycolytic flux and the production of proinflammatory cytokines [43]. However, other modulations of PFKFB3 activity (including acetylation, methylation, S-glutathionylation and ubiquitination) have not been reported in the infectious state. This could be a potential point of investigation for sepsis.

### Modulation of immunocyte inflammatory activation

Metabolic routes determine the fate of immunocytes and influence their inflammatory response ability. Metabolic reprogramming is the core process underlying immunocyte activation in inflammatory states [53]. Enhancement of glycolysis is one of the common metabolic features of many immunocytes during sepsis. Accumulated evidence has revealed that PFKFB3-driven glycolysis is the key link in the development, differentiation and activation of immunocytes, including macrophages [54–56], neutrophils [22], dendritic cells [57], lymphocytes [58–60], and myeloid-derived suppressor cells [61]. Of these cells types, most published studies are on the role of PFKFB3-driven glycolysis in the polarization of macrophages in sepsis. In addition, there is a new research focus on the role of PFKFB3-driven glycolysis in neutrophil inflammatory activation in immune metabolism and sepsis. No studies have been reported on PFKFB3-driven glycolysis and other immunocytes in sepsis. Therefore, we will review in detail the studies related to PFKFB3-driven glycolysis in macrophage polarization and neutrophil activation in sepsis.

### Macrophage inflammatory activation

Macrophages are highly plastic innate immune cells and play a key role in the pathophysiology of sepsis [62]. Macrophages can be polarized into M1 (inflammatory phenotype) macrophages and M2 (anti-inflammatory phenotype) macrophages in response to changes in the environment [63]. During the early stage of sepsis, macrophages undergo M1 differentiation, which contributes greatly to the hyperinflammation stage by releasing multiple proinflammatory cytokines [64–66]. However, excessive inflammatory M1 macrophage activation may lead to the development of organ dysfunction [67–69]. Therefore, modulating the inflammatory activation of macrophages (especially the M1 subtype) can decrease the septic mouse mortality rate by ameliorating inflammation [70–72].

Accumulated evidence has revealed that macrophages adopt distinct metabolic characteristics that correlate with their functional state, which is known as metabolic reprogramming [73]. Under physiological conditions, oxidative phosphorylation (OXPHOS) is the major metabolic pathway for macrophages [74]. During infection, M1 macrophages shift their metabolic profile from OXPHOS to glycolysis to enable them to meet the energy demands of a rapid inflammatory response [9,56,75–77]. Consistent with this viewpoint, many studies have confirmed that M1 macrophages exhibit enhanced glycolysis [78–81], while pharmacological inhibition of macrophage glycolysis reduces their phagocytic activity and cytokine production [73,82–84].

**Figure 4.**
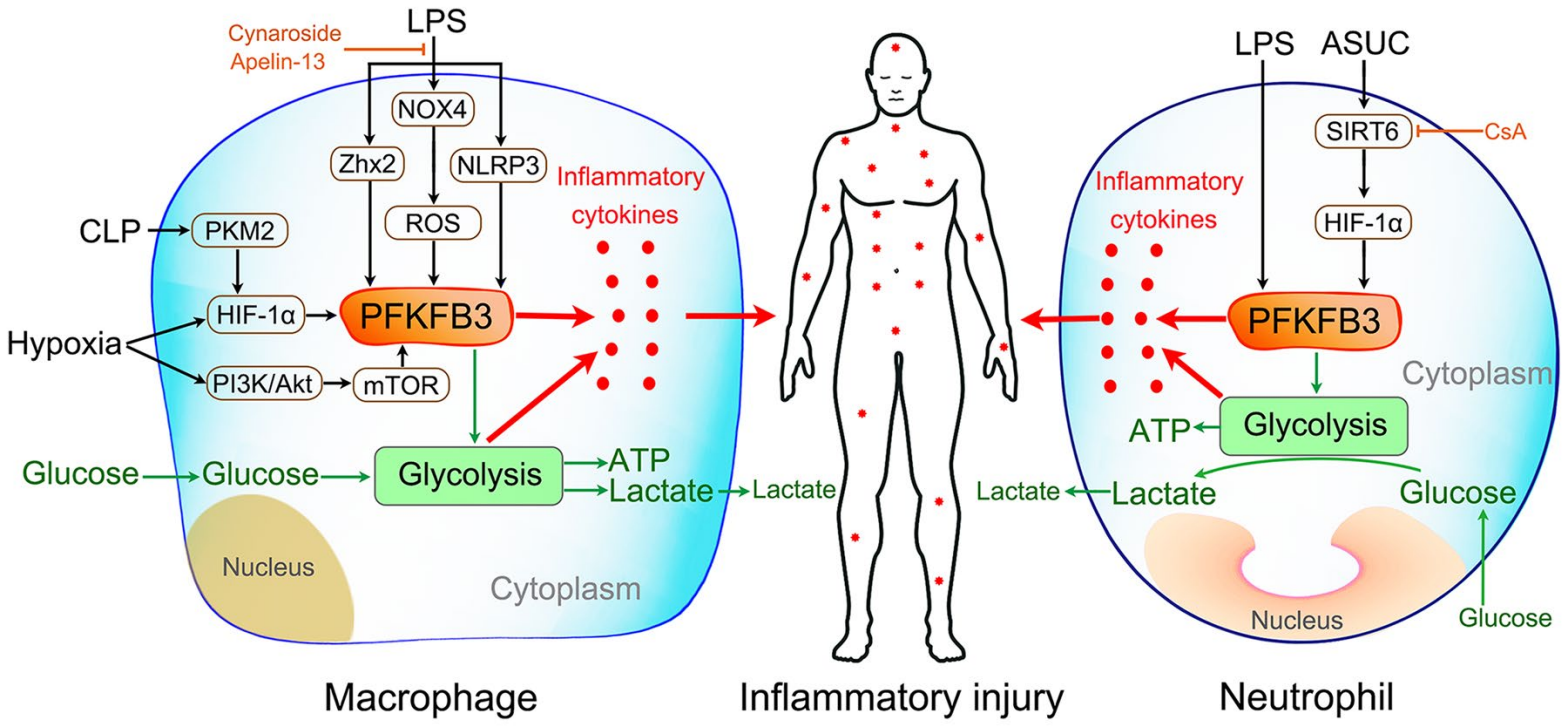
Modulation of macrophage (left) and neutrophil (right) inflammatory activation. LPS stimulation, cecal ligation and puncture (CLP) challenge, and a hypoxic state can promote the upregulated expression of PFKFB3 in macrophages *via* different signaling pathways. Then, the upregulated PFKFB3 increases macrophage inflammatory cytokine production by driving glycolysis. Similar results were found in LPS-stimulated neutrophils. However, inhibiting PFKFB3-driven glycolysis can effectively suppress neutrophil inflammatory activation. In addition, cyclosporine A alleviates acute severe ulcerative colitis (ASUC) by promoting neutrophil HIF-1α expression and restricting excessive neutrophil activation in the SIRT6-HIF-1α-glycolysis axis.

Various studies have revealed that LPS stimulation promotes the upregulated expression of PFKFB3 in macrophages, which contributes to the increased flux of glycolysis and production of inflammatory cytokines (Figure 4) [22,51,56,85,86]. Similar results were found in cecal ligation and puncture (CLP)-mice [87]. Further studies showed that LPS upregulated PFKFB3, which was shown to be involved in NLRP3 inflammasome activation [56], NOX4-dependent ROS production [85], zinc finger and homeobox (Zhx2) activation [86], and PKM2/HIF-1α axis transmission (Figure 4) [87]. It has also been revealed that LPS induces classical microglial activation and proinflammatory effects *via* HIF-1α/PFKFB3 signaling cascades (Figure 4) [88]. A recent study showed that hypoxia, a common pathophysiological change in sepsis, induces enhanced PFKFB3-involved glycolysis in macrophages, and the mechanism involved the PI3K/Akt/mTOR and HIF-1α signaling cascades (Figure 4) [43]. All of these results indicated that PFKFB3-driven macrophage glycolysis is a potential therapeutic target for sepsis. Indeed, different attempts to explore this target have emerged over the past two years.

In 2021, Xu and colleagues revealed that a deficiency in myeloid *Pfkfb3* protects mice from lung edema and cardiac dysfunction in LPS-induced endotoxemia [22]. The mechanism involves attenuating LPS-induced glycolytic flux and subsequently suppressing proinflammatory gene expression. In the same year, Pei and colleagues found that cynaroside, a flavonoid compound, mitigates sepsis-induced liver injury by inhibiting PFKFB3-driven glycolytic metabolism and then preventing macrophage polarization into the M1 phenotype [87]. In 2022, Yuan and colleagues revealed that apelin-13, an endogenous ligand for angiotensin type 1 receptor-associated protein, protects against LPS-induced inflammatory responses and acute lung injury by inhibiting PFKFB3-driven macrophage glycolysis [85]. All of the above attempts suggest that inhibiting PFKFB3-involved glycolytic metabolism can reduce sepsis-induced organ damage. However, a recent study indicated that berberine protected mice against *Salmonella typhimurium* infection and endotoxic shock by inducing PFKFB3-driven aerobic glycolysis and modulating cytokine responses in macrophages [51]. In addition, evidence indicates that PFKFB3-driven macrophage glycolysis is a crucial component of the body’s immune system’s resistance to aspergillosis [89] and viral [90] and *Francisella tularensis* [91] infections.

In conclusion, sepsis accelerates PFKFB3-driven glycolysis in macrophages. The accelerated glycolysis then promotes proinflammatory macrophage polarization and inflammatory activation. Subsequently, the activated proinflammatory macrophages induce inflammatory injury by releasing proinflammatory factors. Therefore, the targeted inhibition of PFKFB3-driven glycolysis in macrophages is a potential therapeutic strategy for avoiding inflammatory injury in sepsis. Of course, this strategy requires further study when infections by certain specific pathogens (including aspergillosis and *Francisella tularensis* and viral infections) are involved.

### Neutrophil inflammatory activation

Neutrophils, the most abundant innate immunocytes (representing 50%–70% of circulating leukocytes), have long been recognized as the first cells to be attracted to infected sites and play an indispensable role in eradicating microbial infections [92]. However, excessive neutrophil activation is also a double-edged sword that results in tissue inflammatory injury and organ dysfunction [93]. Recent studies have revealed that neutrophils adapt to their activated state by enhancing glycolysis (commonly known as glycolytic reprogramming) [94–96]. Therefore, efforts to understand neutrophil glycolytic reprogramming may provide new clues for developing better therapeutics for sepsis.

Glycolysis is the predominant metabolic pathway of mature neutrophils, as they contain relatively few mitochondria (5–6 on average), which participate in adenosine triphosphate (ATP) synthesis at only a very low level [94,97–100]. Several well-executed studies have revealed that glycolysis is the main fuel source for neutrophils to perform their immune functions, including chemotaxis, phagocytosis, and neutrophil extracellular trap (NET) formation [101–103]. In 2021, Lu and colleagues revealed a novel mechanism of cyclosporine A in alleviating acute severe ulcerative colitis (ASUC) by promoting neutrophil HIF-1α expression and restricting excessive neutrophil activation in the SIRT6-HIF-1α-glycolysis axis (Figure 4) [104]. In the same year, our study first revealed that the expression of PFKFB3 was significantly increased in LPS-challenged and septic neutrophils (Figure 4) [22]. We also found that inhibition of PFKFB3 activity and acceleration of *Pfkfb3* gene degradation can effectively suppress neutrophil inflammatory activation. Therefore, targeting neutrophil PFKFB3-driven glycolysis is a potential therapeutic strategy to modulate neutrophil functions in sepsis.

### Modulation of nonimmune cell activation

Recently, nonimmune cells, usually considered structural cells, are expected to be considered regulators and effectors in the host’s defense system [105–107]. Damage to nonimmune cells is the fundamental cause of inflammatory injury to tissues and organs [108]. Accumulated evidence has revealed that PFKFB3-driven glycolysis is also observed in nonimmune cells, such as endothelial cells (ECs) [109], lung fibroblasts [24], epidermal keratinocytes [110], mesenchymal cells [111], and hematopoietic cells [112]. EC dysfunction is one of the common characteristics of sepsis-induced inflammatory injury [6]. Lung fibroblast dysfunction is the main cause of sepsis-related pulmonary fibrosis [113]. In recent years, several published studies have indicated that PFKFB3-driven glycolysis reprogramming plays an important role in sepsis-induced EC and lung fibroblast damage. In this section, we will review in detail the studies related to PFKFB3-driven glycolysis and sepsis-induced damage to ECs and lung fibroblasts.

### EC inflammatory responses

ECs are usually located on the inner wall of blood vessels and control the localization of the balance of proinflammatory and anti-inflammatory responses [114]. EC activation and dysfunction are involved in sepsis, which is strongly associated with multiple-organ dysfunction and high mortality [6,115,116]. Recent studies have revealed that ECs mainly rely on glycolysis rather than OXPHOS to generate ATP to support cellular functions and activation [117–119]. As a key regulator of glycolysis, PFKFB3 is highly expressed in ECs and promotes endothelial cell migration [119]. Furthermore, studies have indicated that PFKFB3-driven glycolysis in ECs mediates the development of vessel formation and angiogenesis [109,119–124]. In addition, the role of PFKFB3-involved glycolysis in sepsis-induced EC dysfunction has gradually emerged in recent years.

**Figure 5.**
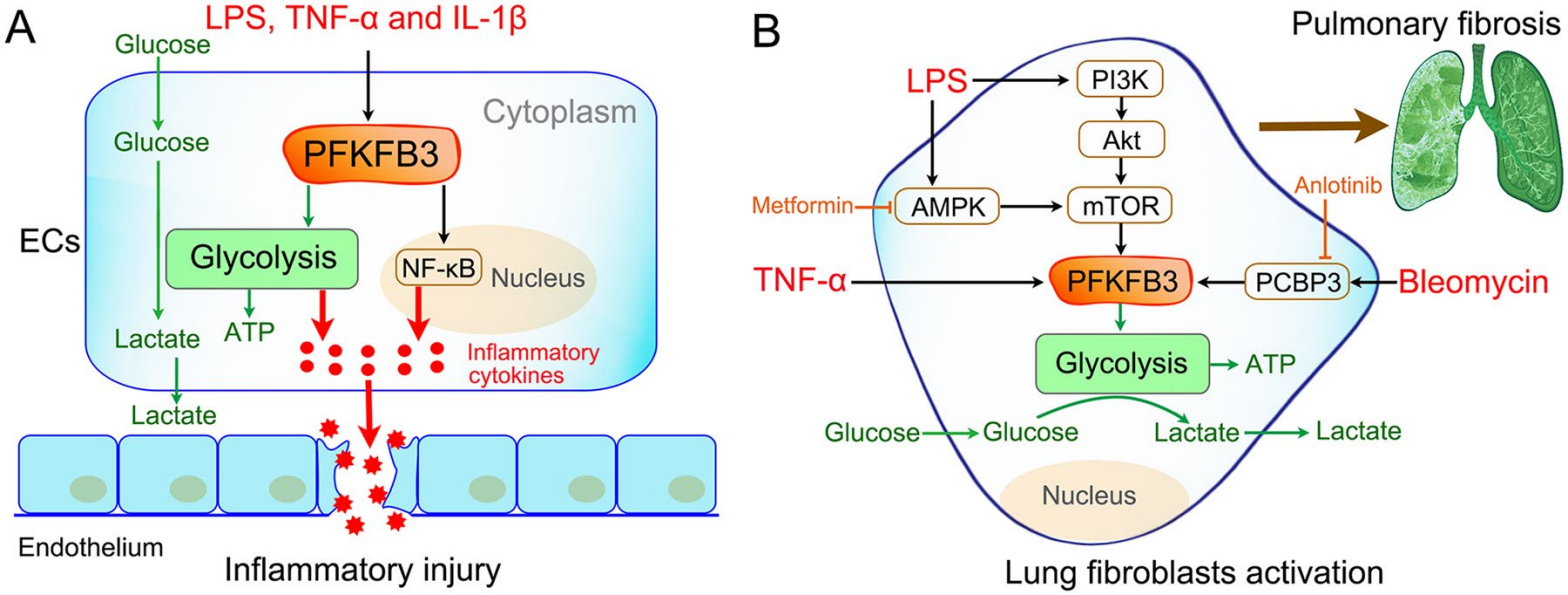
Modulation of nonimmune cell activation. (A) Inflammatory activation of endothelial cells (ECs). Well-known inflammatory stimuli (such as LPS, TNF-α and IL-1β) promote the upregulated expression of PFKFB3 in ECs. Then, the upregulated PFKFB3 increases EC inflammatory cytokine production by driving glycolysis. The mechanism may involve nuclear factor κB (NF-κB) pathway activation. (B) Lung fibroblast activation and proliferation. LPS promotes lung fibroblast activation and proliferation by enhancing PFKFB3-driven glycolysis via different signaling pathways, such as the PI3K-Akt-mTOR pathway and AMPK-mTOR pathway. Similar results were found after bleomycin and TNF-α stimulation. Inhibition of PFKFB3-driven glycolysis in fibroblasts reverses bleomycin/LPS-induced pulmonary fibrosis.

Well-known inflammatory stimuli (such as LPS, TNF-α and IL-1β) have been reported to markedly increase PFKFB3-driven glycolysis in ECs (Figure 5(A)) [23,125–127]. Consistent with increased glycolytic flux, the inflammatory response of ECs was also enhanced. However, inhibition of PFKFB3 resulted in reduced endothelial proinflammatory responses [23]. Furthermore, ablation of the endothelial *Pfkfb3* gene protects mice from acute lung injury in LPS-induced endotoxemia [126]. Mechanistically, the effects of PFKFB3 on EC inflammatory activation may involve the nuclear factor κB (NF-κB) pathway (Figure 5(A)) [23,126]. Thus, all of these studies demonstrate that PFKFB3-driven glycolysis plays a critical role in sepsis-induced endothelial inflammation. Targeting PFKFB3-driven EC glycolysis is an efficient therapeutic strategy for sepsis.

### Lung fibroblast activation and proliferation

Pulmonary fibrosis is a serious lung disease that is characterized by overactivation and proliferation of lung fibroblasts and excessive collagen deposition [128]. Experimental and clinical studies demonstrate that sepsis may trigger the development of persistent pulmonary fibrosis, which contributes to the high mortality rates in septic patients [129–131]. Exploring the mechanism of sepsis-related pulmonary fibrosis and finding a potential intervention approach for the prevention and treatment of septic lung injury have become research hotspots.

Recent evidence indicates that pulmonary fibrosis is closely associated with enhanced aerobic glycolysis in lung fibroblasts [132–135]. Glycolysis is necessary not only for fibroblast growth and proliferation but also for the regulation of fibroblast activation and migration [136–138]. A previous study reported that inhibition of glycolysis by the PFKFB3 inhibitor 3PO or genomic disruption of the *Pfkfb3* gene blunted the differentiation of lung fibroblasts into myofibroblasts and attenuated profibrotic phenotypes in myofibroblasts [139]. Xu and colleagues demonstrated that LPS promotes collagen synthesis in lung fibroblasts through aerobic glycolysis *via* the activation of the PI3K-Akt-mTOR/PFKFB3 pathway [44]. In 2022, the team also revealed that LPS-induced macrophage secretion of TNF-α could initiate fibroblast PFKFB3-driven aerobic glycolysis, which plays an essential role in LPS-induced pulmonary fibrosis [24].

In addition, some drugs have been reported to inhibit pulmonary fibrosis by interfering with PFKFB3‑associated glycolysis. In 2021, Tang and colleagues found that metformin, a biguanide anti‑hyperglycemic agent, prevents LPS‑induced collagen synthesis in lung fibroblasts by regulating PFKFB3‑mediated aerobic glycolysis [140]. A mechanistic study showed that metformin affects the molecular activity of PFKFB3 by regulating the AMPK/mTOR signaling pathway. In addition, Chen and colleagues observed that anlotinib, a novel multitargeted tyrosine kinase inhibitor, exerts potent antifibrotic effects by inhibiting PFKFB3-driven glycolysis in myofibroblasts [141]. Furthermore, they observed that anlotinib inhibited PFKFB3-driven by downregulating PCBP3, a post-transcriptionally regulated RNA-binding protein. All of these findings indicate that targeting PFKFB3‑driven glycolysis in lung fibroblasts might be a promising therapeutic approach for septic pulmonary fibrosis.

## Conclusion

Under physiological conditions, PFKFB3 is expressed at low levels in a wide variety of cells and is responsible for stimulating glycolysis through the allosteric activation of PFK-1. It is essential for cell growth, differentiation and function. In sepsis, PFKFB3 is rapidly (approximately 6 h after LPS stimulation) increased and phosphorylated, which contributes to the rapidly increased glycolytic flux and subsequent inflammatory injury. On the one hand, PFKFB3-derived glycolysis promotes inflammatory activation of immune cells (macrophages and neutrophils), which induces inflammatory injury by releasing proinflammatory factors. On the other hand, it can induce inflammatory injury in ECs and promote lung fibroblast proliferation. Inhibition of PFKFB3 has additionally shown great potential in reducing inflammatory damage and improving the prognosis of sepsis. Therefore, efforts to understand PFKFB3 may provide a novel combinatorial therapeutic target for the effective treatment of sepsis.

However, many questions remain to be clarified in detail. For example, it is still not known whether the metabolic timelines of PFKFB3-driven glycolysis in different immune and nonimmune cells in sepsis are consistent. In addition, we do not know whether there is some crosstalk between immune or nonimmune cells based on PFKFB3-driven glycolysis. All of these factors should be further studied in the future.

## Data Availability

Data sharing does not apply to this article, as no new data were created or analyzed in this study.
